# Socioeconomic inequality in compliance with precautions and health behavior changes during the COVID-19 outbreak: an analysis of the Korean Community Health Survey 2020

**DOI:** 10.4178/epih.e2022013

**Published:** 2022-01-09

**Authors:** Ga Bin Lee, Sun Jae Jung, Yang Yiyi, Jea Won Yang, Hoang Manh Thang, Hyeon Chang Kim

**Affiliations:** 1 Department of Preventive Medicine, Yonsei University College of Medicine, Seoul, Korea; 2 Department of Public Health, Yonsei University Graduate School, Seoul, Korea

**Keywords:** Public health, Educational status, Income, Coronavirus, Health behavior

## Abstract

**OBJECTIVES:**

This study examined socioeconomic inequalities in compliance with precautions and health behavior changes during the coronavirus disease 2019 (COVID-19) outbreak using a representative Korean sample.

**METHODS:**

This exploratory study utilized around 210,000 participants aged ≥25 years in the Korean Community Health Survey 2020. Socioeconomic status was measured with educational attainment and household income. Outcomes included non-compliance with 8 precaution measures and deterioration in 6 health behaviors. The relative inequality index (RII) was calculated to quantify the degree of inequality by education and income level. RII values >1.0 indicate that deprived people have a higher frequency of health problems, and RII values <1.0 conversely indicate a higher frequency of health problems in more advantaged groups.

**RESULTS:**

People with lower education or income levels tended to have higher rates of non-compliance with COVID-19 safety precautions (RII range, 1.20 to 3.05). Lower education and income levels were associated with an increased smoking amount (RII=2.10 and 1.67, respectively) and sleep duration changes (RII=1.21 and 1.36, respectively). On the contrary, higher education and income levels were associated with decreased physical activity (RII=0.59 and 0.77, respectively) and increased delivery food consumption (RII=0.27 and 0.37, respectively). However, increased alcohol drinking was associated with lower education and income levels in younger men (RII=1.73 and 1.31, respectively), but with higher levels in younger women (RII=0.73 and 0.68, respectively).

**CONCLUSIONS:**

Our findings suggest the need to develop customized strategies, considering the characteristics of the target population, to decrease the burden and impact of the COVID-19 outbreak.

## GRAPHICAL ABSTRACT

[Fig f3-epih-44-e2022013]

## INTRODUCTION

The coronavirus disease 2019 (COVID-19) outbreak was first reported in December 2019 and continued to spread worldwide [1]. In response to the COVID-19 outbreak, worldwide governments have imposed mask-wearing and social distancing to mitigate the pandemic [2]. Specifically, the Korean government implemented a strong national social distancing campaign; people were asked to leave their houses only when necessary, and many community facilities were closed, with the potential for non-compliant facilities to receive administrative orders [3]. Additionally, in Korea, isolation of confirmed cases and self-quarantine of contacts have been the norm [4]. These policies have been helpful in reducing or slowing the COVID-19 epidemic [5]. However, strengthening quarantine measures and social distancing have worsened health behavior and increased psychological stress [6,7]. Socioeconomic characteristics may affect the degree of compliance with COVID-19 prevention measures and deterioration of health behavior [8], as well as age or comorbidities [9]. Although there are some differences according to types of behaviors or socioeconomic status (SES) indicators, usually, people with lower SES are more likely to engage in deleterious health behaviors [10,11] and less likely to receive information on safety and health precautions [12]. During pandemics, economic hardships may affect lower-SES people’s access to material resources and health services [13]; accordingly, the already existing-socioeconomic inequality in health behaviors may worsen. During the COVID-19 pandemic, several studies have reported differences in health and safety behaviors according to SES [14,15]. However, the previous studies cannot be generalized due to their limited sampling methods, size, and characteristics. Therefore, we analyzed a Korean representative sample to explore SES inequalities in compliance with safety precautions and health behavior changes during the COVID-19 outbreak.

## MATERIALS AND METHODS

### Data and sample

We analyzed an anonymized research dataset obtained from the Korean Community Health Survey (KCHS) for 2020. The KCHS is a nationwide survey of health interviews conducted by the Korea Disease Control and Prevention Agency (KCDA), aiming to establish a standardized community survey with which to develop health projects for all local districts. Detailed information on the study design and aims of the KCHS has been previously reported [16]. Briefly, before each year’s interview, sample households are selected to represent each of the administrative districts of Korea. Based on registration data from the Ministry of the Interior and Safety and data on housing types from the Ministry of Land, Infrastructure, and Transport, sample households are selected. On average, 900 individuals are examined by each health center in 255 local districts, yielding about 230,000 people in total. Trained interviewers conduct one-to-one interviews with individuals aged 19 years or older. The KCHS is the only data source that collects standardized health information from all administrative districts in Korea. In the 2020 KCHS, all interviewers completed a COVID-19 polymerase chain reaction test and vaccinations before the start of the survey. Interviewers checked their health status every day, and those with symptoms did not participate in the interview. Additionally, while visiting households, interviewers strictly followed precautions including wearing a mask, sanitizing hands, checking body temperature, and maintaining physical distance from the participant [17]. In the 2020 KCHS, questions about the degree of compliance with safety measures to prevent COVID-19 and changes in health behavior after the COVID-19 outbreak were added. In current study, we excluded participants aged under 25 years since they may not have finished their education or be economically inactive [18]. The final study sample consisted of 214,703 individuals for the analysis using educational attainment and 209,455 individuals for the analysis using household income in 2020. A flow chart of the selection of study participants is presented in Supplementary Material 1.

### Measurements

Educational attainment was categorized into 4 groups: elementary school or less, middle school, high school, and college or more (reference group). Equivalized household income was defined as the total monthly household income divided by the square root of the number of household members. Participants were divided into 4 groups according to the quartiles of equivalized household income, from Q1 (lowest) to Q4 (highest, reference group). The quartiles of monthly household income were separately calculated by gender and age (< 65 or ≥ 65) considering that those aged ≥ 65 were mostly economically inactive. Self-reported responses for compliance with COVID-19 safety precautions and deterioration of health behaviors during the COVID-19 outbreak were utilized as the main outcomes. First, compliance with COVID-19 safety precautions was evaluated through questions asking whether the participants, in the last week, (1) covered their mouth while coughing; (2) practiced regular disinfection; (3) practiced regular ventilation; (4) wore a mask in indoor facilities such as public transportation, restaurants, and department stores; (5) wore a mask when it was hard to keep a distance; (6) adhered to recommendations on the minimal physical distance (2 meters); (7) refrained from visiting patients in hospitals; and (8) refrained from going out. These items had responses of “completely complied,” “complied,” “failed to comply,” or “not applicable.” Participants were dichotomized according to responses of “failed to comply.” Health behavior changes were evaluated using questions asking whether participants experienced changes in (1) physical activity including walking or exercise; (2) sleep duration; (3) amount of instant meal and soda consumption; (4) amount of delivery food consumption; (5) alcohol drinking; and (6) amount of smoking. These questions had responses of “increased,” “did not change,” “decreased,” or “not applicable.” Health behavior deterioration was captured as increases in the smoking amount, alcohol drinking, consumption of instant food and soda, or consumption of delivery food; changes in sleep duration; and decreases in physical activity.

### Statistical analysis

The demographic characteristics of the participants were presented as weighted mean± standard deviation or frequency and weighted proportion. Logistic regression models were used to estimate the associations between SES (educational attainment or household income) and outcomes of interest (failure to comply with precautions and health behavior deterioration). Age-adjusted odds ratios (ORs) and 95% confidence intervals (CIs) were calculated using the PROC SURVEYLOGISTIC procedure to apply stratification, primary sampling units, and population weights. Additionally, the analysis of compliance with precautions was adjusted for COVID-19-related characteristics including quarantine/isolation experience due to COVID-19 infection and recent experience of fever/coughing. For health behavior changes, smoking status (past/current), alcohol drinking frequency, sleep duration, and moderate physical activity (yes/no) were additionally adjusted in the models for increased smoking amount, increased alcohol drinking, changed sleep duration, and decreased physical activity. Furthermore, the age-adjusted frequencies of non-compliance with precautions and health behavior deterioration were estimated using the direct standardization method based on the 2015 Korean census.

The relative index of inequality (RII) was developed to quantify the relative inequality gap between those who are positioned in the lowest and the highest SES categories [19]. The RII indicates the ratio between the most and least disadvantaged groups. If it is equal to 1.0, there is no inequality, higher values indicate worse outcomes in the most disadvantaged group, and values less than 1.0 indicate worse outcomes in the most advantaged group [20]. In the current study, RIIs by education or income levels were separately calculated. First, to estimate RII by education level, the relative position of educational attainment was obtained from rescaling the categorical educational attainment variables to have a continuous range from 0 (the highest education level) to 1 (the lowest education level). The relative position of educational attainment was assigned to each category based on the proportion of participants above the midpoint in the category. For instance, if 10% of participants were included in the highest education level group, they were assigned a relative position of 0.05 (0.0+0.5× 0.10). If 30% of participants were included in the next highest education level group, a relative position of 0.25 (0.1+0.5× 0.30) was assigned, and so forth. Second, this relative position was treated as an independent variable in binomial regression models, assuming a linear association between education levels and outcomes. Lastly, the coefficient of the relative position was the RII value by education level. RIIs by income levels were also calculated using the same process. All analyses were conducted in the total population and then stratified by gender and age group (< 65 or ≥ 65). All statistical analyses were performed using SAS version 9.4 (SAS Institute Inc., Cary, NC, USA) and R version 4.1.2 (R Foundation for Statistical Computing, Vienna, Austria). A 2-sided p-value less than 0.05 was considered to indicate statistical significance.

### Ethics statement

The data in this study were obtained from the KCHS, KDCA, Ministry for Health and Welfare in Korea. Our data from the KCHS are freely available if researchers submit appropriate institutional review board clearance to the KDCA. This study was approved by the Institutional Review Board of Severance Hospital at Yonsei University College of Medicine (IRB No: 4-2021-1256).

## RESULTS

Table 1 presents the sociodemographic characteristics of all study participants and by gender and age groups. Overall, women had higher proportions of lower education levels (e.g., elementary or middle school graduates) than men in both age groups. Additionally, more than half of men aged < 65 were manual workers (32.3%) or professional administrators (19.5%), while most women aged < 65 were homemakers (39.2%) or sales workers (18.2%). In the elderly (age ≥ 65) group, both men and women were mostly unemployed or homemakers (men: 61.6%; women: 77.7%) or manual workers (men: 19.8%; women: 10.8%).

Table 2 presents ORs for failure to comply with safety precautions and health behavior deterioration according to educational attainment or household income. Overall, participants with lower SES tended to have higher odds of failure to comply with COVID-19 safety precautions than the highest SES group. In health behavior deterioration, the lower SES groups tended to have higher ORs for changes in sleep duration and increased smoking amount, while they had lower ORs for decreased physical activity, increased instant/delivery food consumption, and increased alcohol drinking. The gender-specific and age-specific results are shown in Supplementary Materials 2-5.

**Figure 1.**
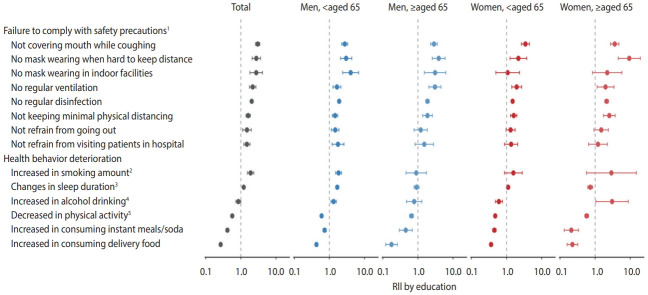
Relative index of inequality (RII) by education levels for failure to comply with coronavirus disease 2019 (COVID-19) safety precautions and health behavior deterioration in the total population and by gender and age. Questions were sorted by descending order of RII values in the total population analysis. ^1^Adjusted for quarantine isolation experience due to COVID-19 infection and recent experience of fever/coughing. ^2^Adjusted for smoking status (current/past). ^3^Adjusted for sleep duration. ^4^Adjusted for alcohol drinking frequencies. ^5^Adjusted for moderate physical activity (yes/no).

**Figure 2.**
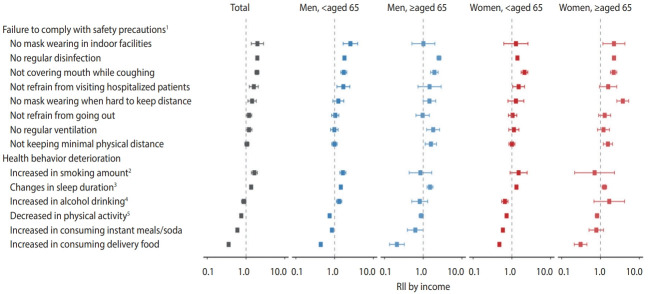
Relative index of inequality (RII) by income levels for failure to comply with coronavirus disease 2019 (COVID-19) safety precautions and health behavior deterioration in the total population and by gender and age. Questions were sorted by descending order of RII values in the total population analysis. ^1^Adjusted for quarantine isolation experience due to COVID-19 infection and recent experience of fever/coughing. ^2^Adjusted for smoking status (current/past). ^3^Adjusted for sleep duration. ^4^Adjusted for alcohol drinking frequencies. ^5^Adjusted for moderate physical activity (yes/no).

Figure 1 shows RIIs by education levels for failure to comply with safety precautions and health behavior deterioration in the total population and by gender and age. Estimates of RIIs were found in Supplementary Material 6. In the total population, the RIIs by education level for failure to comply with COVID-19 safety precautions were greater than 1.0, indicating that less-educated people were more likely to fail to comply with safety precautions than the more-educated people. However, in women, these educational differences in failure to comply with several precautions tended to be weaker; the RIIs for not mask-wearing in indoor facilities, not refraining from going out, and not refraining from visiting hospitalized patients were not significant in women. In health behavior deterioration, the RIIs in the total population showed different directions according to the type of health behavior. Increased smoking amount and sleep duration changes (RII= 2.10 and 1.21, respectively) were prominent in less-educated people, while decreased physical activity, increased consumption of instant meals, and increased consumption of delivery food were prominent in more-educated people (RII= 0.59, 0.42, and 0.27, respectively). In particular, the tendency for less-educated people to experience worse changes in smoking and sleep duration were most prominent in younger men (RII = 2.06 for increased smoking; RII = 1.69 for changed sleep duration) and younger women (RII= 3.35 for increased smoking; RII= 1.11 for changed sleep duration) but not in elderly men and women. Furthermore, deterioration in alcohol drinking was apparent in groups with different education levels by gender; it was associated with lower education levels in younger men (RII= 1.73), but with higher education levels in younger women (RII = 0.73). Forest plots and estimates of RIIs by income levels are presented in Figure 2 and Supplementary Material 7, respectively, and the overall results are similar to those of RIIs by education levels.

## DISCUSSION

Overall, in the nationwide health survey conducted in 2020, significant socioeconomic discrepancies were observed in compliance with COVID-19 precautions and in health behavior changes. People with lower education or lower income levels were more likely to fail to comply with safety precautions, but the relative degree of inequality differed by types of precaution measure and by respondents’ gender and age. Socioeconomic discrepancies in health behavior deterioration showed variation not only in their magnitude, but also in the direction of inequality indicators.

Our findings of SES inequalities in safety precautions align with those of a previous study analyzing the data of 6,000 individuals from the United States, the United Kingdom, Italy, China, Japan, and Korea, in that high-income people adopted more self-protective behaviors such as mask-wearing during the COVID-19 pandemic [15]. In the current study, although the overall rate of compliance with safety measures and social distancing for the prevention of COVID-19 was very high, the compliance rate was different according to SES. In particular, the compliance rate for wearing masks in indoor facilities or when it was hard to keep distance and for regular ventilation was very high, ranging from 95% to 98%, in all education/income groups (Supplementary materials 8-11). Nevertheless, the RII by education level was greater than 1.0 for all 8 precaution measures, and the RII by income level was also significantly greater than 1.0 for 7 out of 8 measures. We used RII, which quantifies the socioeconomic gradient in relative terms, to evaluate socioeconomic inequalities in the frequency of failure to comply with precaution measures. However, when making policy decisions, absolute estimates are also needed to evaluate the cost-effectiveness of policy alternatives [21]. The observed socioeconomic inequalities in precaution compliance may amplify the risk of COVID-19 infection among people with low SES, considering that deprived people are easily exposed to vulnerable environments. Moreover, the link between low education levels and non-compliance with COVID-19 safety precautions was more clearly observed in men under age 65, most of whom were employed workers. Socially or economically deprived people are more likely to live in worse living conditions or have jobs that do not allow telecommuting [13]. Therefore, the socioeconomic inequality that presents in COVID-19 safety precautions or social distancing needs to be considered more seriously when making policy decisions.

Socioeconomic inequalities were also observed in deteriorating health behavior after the COVID-19 outbreak, but the direction and magnitude were different depending on the type of health behavior. Our study findings align with those of a previous study with 1,809 United States participants recruited through convenience sampling, which reported that people with higher educational attainment tended to decrease tobacco use or increase alcohol consumption during the COVID-19 outbreak [14]. In the current study, an increased smoking amount was observed more frequently in both groups with lower education and income levels, but disproportionate changes in sleep duration were found only in those with lower income levels. Unexpectedly, increased alcohol drinking, decreased physical activity, and increased consumption of unhealthy food were observed more in people with higher education or income levels. This is probably due to differences in the baseline status of health behaviors, according to which people with higher SES had better lifestyles before the COVID-19 outbreak. Since an increase in smoking amount would usually occur only among smokers, such baseline effects would not have an impact on smoking behaviors. In addition, people with higher SES have encountered more changes in working patterns such as telecommuting [13] and may have been more affected by the closure of sports facilities [22]. Reasons for the greater increase in consumption of delivery/instant food among higher SES groups may include more frequent telecommuting and the lower economic barriers to buying such foods.

In the current study, socioeconomic inequalities in increases in smoking amounts and changes in sleep duration were more prominent in the younger group. Among young people, who are likely to be economically active, those with lower education or income levels showed greater fear of financial difficulties during COVID-19 [13,23]. Studies have also reported that psychological stress associated with COVID-19 exacerbates sleep quality and increases smoking volume [24,25]. Interestingly, increased alcohol drinking showed different patterns in younger men and women. The increase in alcohol consumption was more prominent in lower SES groups in younger men, but more prominent in higher SES groups in younger women. This finding partially aligns with the results of a previous study using international data from 15 countries in Europe and South America; heavy drinking was found in more-educated women and less-educated men [26]. However, the age-specific and gender-specific impact of COVID-19 on alcohol consumption needs to be further studied.

An advantage of our study is that it used a representative sample that covered all administrative districts of Korea and used data collected according to a standardized questionnaire and quality control program. However, several limitations should be considered. First, there is a possibility of measurement error as both compliance with COVD-19 precautions and changes in health behaviors were measured based on self-report surveys. We cannot completely rule out the possibility that these measurement errors may have varied depending on the socioeconomic level. Second, our study could be considered as an exploratory analysis in the association of SES with precaution compliance and health behavior changes. Confirmatory studies are required to further evaluate socioeconomic inequalities. Third, the RII is a relative indicator of inequality and is useful for comparing the degree of inequality between different health outcomes or subgroups, but these findings need to be supplemented with data on absolute inequality levels.

In conclusion, this study of a representative Korean sample found that socioeconomic inequalities existed in compliance with COVID-19 precautions and in health behavior deterioration. People with low SES were more likely to fail to comply with safety precautions, but the magnitude of inequality varied by gender, age, and type of precaution measure. The socioeconomic inequality in health behaviors differed not only in its magnitude, but also in the direction of inequality indicators. Our findings suggest that it is necessary to develop a customized strategy that considers the characteristics of the target population to reduce the burden and impact of the COVID-19 outbreak.

## Figures and Tables

**Figure f3-epih-44-e2022013:**
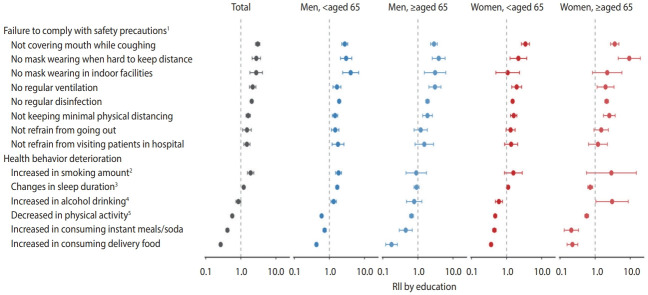


**Table 1.** Demographic characteristics of participants by gender and age in the Korea Community Health Survey 2020

Characteristics		Total (n=214,966)	Men (n=97,250)		Women (n=117,716)	
			Age <65 (n=66,924)	Age ≥65 (n=30,326)	Age <65 (n=75,230)	Age ≥65 (n=42,486)
Age (yr)		51.49±0.05	44.90±0.05	73.39±0.05	45.65±0.05	74.17±0.04
Educational attainment						
	Elementary or less	53,785 (13.7)	3,075 (2.6)	12,451 (31.9)	6,672 (5.0)	31,587 (63.7)
	Middle school	24,825 (8.8)	5,119 (5.1)	6,353 (20.3)	8,047 (7.4)	5,306 (15.7)
	High school	67,002 (33.2)	26,078 (35.8)	7,702 (30.1)	29,028 (37.0)	4,194 (15.2)
	College or higher	69,091 (44.3)	32,577 (56.5)	3,771 (17.6)	31,393 (50.6)	1,350 (5.4)
Monthly household income						
	Q1 (lowest)	63,353 (24.5)	19,916 (25.0)	8,759 (23.8)	22,114 (24.7)	12,564 (23.0)
	Q2	53,417 (24.5)	15,300 (23.9)	7,829 (24.6)	18,886 (25.3)	11,402 (24.2)
	Q3	43,256 (21.9)	12,277 (20.3)	7,361 (26.5)	13,906 (20.8)	9,712 (26.8)
	Q4 (highest)	49,429 (29.1)	17,436 (30.9)	5,854 (25.1)	18,119 (29.2)	8,020 (26.0)
Job						
	Professional administrator	22,244 (14.5)	10,817 (19.5)	817 (3.9)	10,418 (16.3)	192 (0.7)
	Office worker	18,925 (12.5)	9,528 (17.3)	308 (1.4)	9,011 (14.3)	78 (0.3)
	Sales worker	27,048 (13.6)	8,577 (14.0)	1,138 (4.0)	15,165 (18.2)	2,168 (5.3)
	Agricultural worker	22,358 (3.2)	5,773 (2.7)	6,846 (9.3)	4,205 (1.5)	5,534 (5.2)
	Manual worker	41,987 (20.1)	22,137 (32.3)	5,178 (19.8)	8,840 (10.5)	5,832 (10.8)
	Other (homemakers or unemployed)	82,198 (36.1)	10,017 (14.2)	16,018 (61.6)	27,528 (39.2)	28,635 (77.7)

Values are presented as weighted mean ± standard deviation or number (weighted proportion).

**Table 2.** Odds ratio for failure to comply with safety precautions and health behavior deterioration during the coronavirus disease 2019 (COVID-19) outbreak according to educational attainment or household income

COVID-19-related questionnaires		Educational attainment (n=214,703)				Household income (n=209,455)			
		College +	High school	Middle school	Elementary or less	Q4 (highest)	Q3	Q2	Q1 (lowest)
Failure to comply with safety precautions^1^									
	Not covering mouth while coughing	1.00 (reference)	1.39 (1.28, 1.51)	1.62 (1.45, 1.80)	2.27 (2.05, 2.50)	1.00 (reference)	1.13 (1.02, 1.25)	1.28 (1.16, 1.42)	1.61 (1.46, 1.77)
	Not performing regular ventilation	1.00 (reference)	1.11 (1.00, 1.24)	1.33 (1.12, 1.58)	2.06 (1.78, 2.39)	1.00 (reference)	1.04 (0.91, 1.18)	0.92 (0.81, 1.05)	1.21 (1.06, 1.37)
	Not performing regular disinfection	1.00 (reference)	1.17 (1.13, 1.20)	1.34 (1.28, 1.40)	1.81 (1.73, 1.89)	1.00 (reference)	1.06 (1.02, 1.11)	1.19 (1.14, 1.23)	1.65 (1.59, 1.73)
	Not mask-wearing in indoor facilities	1.00 (reference)	1.20 (0.97, 1.50)	1.52 (1.12, 2.07)	2.19 (1.64, 2.93)	1.00 (reference)	1.54 (1.16, 2.04)	1.27 (0.95, 1.68)	1.87 (1.39, 2.52)
	Not mask-wearing when hard to keep distance	1.00 (reference)	1.28 (1.08, 1.50)	1.62 (1.30, 2.02)	2.11 (1.75, 2.54)	1.00 (reference)	1.10 (0.90, 1.34)	1.01 (0.83, 1.22)	1.34 (1.11, 1.62)
	Not keeping the minimum recommended physical distance	1.00 (reference)	1.13 (1.06, 1.22)	1.29 (1.16, 1.45)	1.45 (1.30, 1.61)	1.00 (reference)	1.05 (0.96, 1.14)	1.03 (0.94, 1.13)	1.05 (0.95, 1.16)
	Not refraining from visiting hospitalized patients	1.00 (reference)	1.29 (1.12, 1.50)	1.22 (0.97, 1.54)	1.26 (1.02, 1.55)	1.00 (reference)	1.13 (0.93, 1.37)	1.23 (1.01, 1.49)	1.42 (1.16, 1.73)
	Not refraining from going out	1.00 (reference)	1.05 (0.95, 1.15)	1.04 (0.89, 1.22)	1.54 (1.33, 1.78)	1.00 (reference)	1.04 (0.93, 1.17)	1.03 (0.91, 1.16)	1.17 (1.03, 1.32)
Health behavior deterioration									
	Decreased physical activity^2^	1.00 (reference)	0.82 (0.79, 0.84)	0.74 (0.71, 0.78)	0.68 (0.65, 0.71)	1.00 (reference)	0.87 (0.84, 0.90)	0.83 (0.80, 0.86)	0.81 (0.78, 0.84)
	Changes in sleep duration^3^	1.00 (reference)	1.18 (1.14, 1.22)	1.24 (1.17, 1.31)	0.99 (0.93, 1.04)	1.00 (reference)	1.01 (0.96, 1.05)	1.14 (1.09, 1.19)	1.23 (1.17, 1.28)
	Increased consumption of instant meals/soda	1.00 (reference)	0.85 (0.82, 0.89)	0.52 (0.47, 0.58)	0.36 (0.32, 0.40)	1.00 (reference)	0.91 (0.87, 0.95)	0.77 (0.73, 0.82)	0.67 (0.62, 0.73)
	Increased consumption of delivery food	1.00 (reference)	0.74 (0.72, 0.77)	0.41 (0.38, 0.45)	0.28 (0.25, 0.30)	1.00 (reference)	0.78 (0.75, 0.81)	0.59 (0.56, 0.62)	0.47 (0.44, 0.51)
	Increased alcohol drinking^4^	1.00 (reference)	1.05 (0.98, 1.12)	0.89 (0.77, 1.01)	0.69 (0.59, 0.81)	1.00 (reference)	0.96 (0.89, 1.04)	0.95 (0.87, 1.04)	0.90 (0.81, 1.00)
	Increased smoking amount^5^	1.00 (reference)	1.34 (1.22, 1.47)	1.36 (1.15, 1.61)	1.36 (1.13, 1.63)	1.00 (reference)	1.13 (1.00, 1.28)	1.26 (1.11, 1.42)	1.44 (1.26, 1.66)

Values are presented as odds ratio (95% confidence interval).

1 Adjusted for quarantine/isolation experience due to COVID-19 infection and recent experience of fever/coughing.

2 Adjusted for moderate physical activity (yes/no).

3 Adjusted for sleep duration.

4 Adjusted for alcohol drinking frequencies.

5 Adjusted for smoking status (current/past).

## SUPPLEMENTARY MATERIALS

Supplementary Material 1. Flow chart of the selection of study participants in Korea Community Health Survey 2020.

Supplementary Material 2. Odds ratio by sex for failure to comply with safety precautions and health behavior deterioration during COVID-19 outbreak according to education attainment in participants aged under 65

Supplementary Material 3. Odds ratio by sex for failure to comply with safety precautions and health behavior deterioration during COVID-19 outbreak according to education attainment in participants aged 65 or more

Supplementary Material 4. Odds ratio by sex for failure to comply with safety precautions and health behavior deterioration during COVID-19 outbreak according to education attainment in participants aged under 65

Supplementary Material 5. Odds ratio by sex for failure to comply with safety precautions and health behavior deterioration during COVID-19 outbreak according to education attainment in participants aged 65 or more

Supplementary Material 6. Estimates of relative index of inequality (RII) assessed by educational attainment failure to comply with precautions and health behavior changes by sex and age

Supplementary Material 7. Estimates of relative index of inequality (RII) assessed by household income for failure to comply with precautions and health behavior deterioration by sex and age

Supplementary Material 8. Age-standardized rates of COVID-19 safety precautions compliance and health behavior deterioration occurrence by educational attainment in men

Supplementary Material 9. Age-standardized rates of COVID-19 safety precautions compliance and health behavior deterioration occurrence by educational attainment in women

Supplementary Material 10. Age-standardized rates of COVID-19 safety precautions compliance and health behavior deterioration occurrence by household income in men

Supplementary Material 11. Age-standardized rates of COVID-19 safety precautions compliance and health behavior deterioration occurrence by household income in women
