# Supplementary material for: Socioeconomic inequality in compliance with precautions and health behavior changes during the COVID-19 outbreak: an analysis of the Korean Community Health Survey 2020
Source: Epidemiol Health. 2022 Jan 9;44:e2022013. doi: 10.4178/epih.e2022013 (PMC8989472; doi:10.4178/epih.e2022013)
Supplement: Supplementary Material 1. — Flow chart of the selection of study participants in Korea Community Health Survey 2020. [file epih-44-e2022013-suppl1.docx]

Supplementary Material 1. Flow chart of the selection of study participants in Korea Community Health Survey 2020.

Enrolled participants in 2020,

n=229,269

(103,894 men and 125,375 women)

Excluded participants age < 25

n=14,303

Participants aged ≥ 25

n=214,966

(97,250 men and 117,716 women)

Household income group:

n=209,455

(94,732 men and 114,723 women)

Educational attainment group:

n=214,703

(97,126 men and 117,577 women)
