# Supplementary material for: Socioeconomic inequality in compliance with precautions and health behavior changes during the COVID-19 outbreak: an analysis of the Korean Community Health Survey 2020
Source: Epidemiol Health. 2022 Jan 9;44:e2022013. doi: 10.4178/epih.e2022013 (PMC8989472; doi:10.4178/epih.e2022013)
Supplement: Supplementary Material 2. — Odds ratio by sex for failure to comply with safety precautions and health behavior deterioration during COVID-19 outbreak according to education attainment in participants aged under 65 [file epih-44-e2022013-suppl2.docx]

| Supplementary Material 2. Odds ratio by sex for failure to comply with safety precautions and health behavior deterioration during COVID-19 outbreak according to education attainment in participants aged under 65 | | | | | | | | | | | | | | | | | | | | | | | | | | | | | | | | | | | |
| --- | --- | --- | --- | --- | --- | --- | --- | --- | --- | --- | --- | --- | --- | --- | --- | --- | --- | --- | --- | --- | --- | --- | --- | --- | --- | --- | --- | --- | --- | --- | --- | --- | --- | --- | --- |
| COVID19-related questionnaires | Men, age < 65 (n=66,849) | | | | | | | | | | | | | | | |  | Women, age < 65 (n=75,140) | | | | | | | | | | | | | | | | | |
|  | College + |  | High school | | | |  | Middle school | | | |  | Elementary or less | | | |  | College + |  | High school | | | |  | Middle school | | | |  | Elementary or less | | | | |  |
|  | OR |  | OR | 95% CI | | |  | OR | 95% CI | | |  | OR | 95% CI | | |  | OR |  | OR | 95% CI | | |  | OR | 95% CI | | |  | OR | 95% CI | | |  |  |
| Failure to comply with safety precautions^1^ |  |  |  |  |  |  |  |  |  |  |  |  |  |  |  |  |  |  |  |  |  |  |  |  |  |  |  |  |  |  |  |  |  |  |  |
| Not covering mouth while coughing | 1.0 |  | 1.49 | (1.33 | - | 1.66) |  | 2.06 | (1.73 | - | 2.46) |  | 2.34 | (1.91 | - | 2.88) |  | 1.0 |  | 1.55 | (1.34 | - | 1.78) |  | 2.03 | (1.66 | - | 2.47) |  | 2.94 | (2.37 | - | 3.67) |  |  |
| No regular ventilation | 1.0 |  | 1.17 | (1.02 | - | 1.33) |  | 1.54 | (1.18 | - | 2.02) |  | 1.92 | (1.37 | - | 2.69) |  | 1.0 |  | 1.18 | (1.00 | - | 1.41) |  | 1.40 | (1.02 | - | 1.93) |  | 2.54 | (1.88 | - | 3.44) |  |  |
| No regular disinfection | 1.0 |  | 1.28 | (1.22 | - | 1.33) |  | 1.59 | (1.46 | - | 1.72) |  | 1.93 | (1.74 | - | 2.15) |  | 1.0 |  | 1.11 | (1.07 | - | 1.16) |  | 1.26 | (1.18 | - | 1.36) |  | 1.57 | (1.45 | - | 1.70) |  |  |
| No mask wearing in indoor facilities | 1.0 |  | 1.56 | (1.19 | - | 2.05) |  | 2.89 | (1.87 | - | 4.46) |  | 3.84 | (2.20 | - | 6.72) |  | 1.0 |  | 0.80 | (0.52 | - | 1.24) |  | 0.80 | (0.43 | - | 1.51) |  | 1.99 | (1.10 | - | 3.59) |  |  |
| No mask wearing when hard to keep distance | 1.0 |  | 1.49 | (1.22 | - | 1.81) |  | 2.17 | (1.54 | - | 3.05) |  | 2.64 | (1.93 | - | 3.61) |  | 1.0 |  | 1.18 | (0.87 | - | 1.61) |  | 1.94 | (1.27 | - | 2.95) |  | 1.87 | (1.24 | - | 2.81) |  |  |
| Not keeping minimal physical distance | 1.0 |  | 1.17 | (1.07 | - | 1.29) |  | 1.35 | (1.12 | - | 1.63) |  | 1.21 | (0.94 | - | 1.55) |  | 1.0 |  | 1.13 | (1.01 | - | 1.26) |  | 1.52 | (1.27 | - | 1.81) |  | 1.52 | (1.25 | - | 1.85) |  |  |
| Not refrain from visiting hospitalized patients | 1.0 |  | 1.38 | (1.12 | - | 1.70) |  | 1.35 | (0.91 | - | 2.02) |  | 1.09 | (0.62 | - | 1.92) |  | 1.0 |  | 1.23 | (0.99 | - | 1.52) |  | 0.96 | (0.65 | - | 1.41) |  | 1.33 | (0.88 | - | 2.01) |  |  |
| Not refrain from going out | 1.0 |  | 1.16 | (1.02 | - | 1.32) |  | 1.10 | (0.87 | - | 1.40) |  | 1.94 | (1.41 | - | 2.67) |  | 1.0 |  | 1.05 | (0.91 | - | 1.22) |  | 1.22 | (0.94 | - | 1.60) |  | 1.45 | (1.08 | - | 1.96) |  |  |
| Health behavior deterioration |  |  |  |  |  |  |  |  |  |  |  |  |  |  |  |  |  |  |  |  |  |  |  |  |  |  |  |  |  |  |  |  |  |  |  |
| Decreased in physical activity^2^ | 1.0 |  | 0.80 | (0.77 | - | 0.84) |  | 0.70 | (0.64 | - | 0.76) |  | 0.69 | (0.61 | - | 0.77) |  | 1.0 |  | 0.76 | (0.73 | - | 0.80) |  | 0.61 | (0.57 | - | 0.66) |  | 0.53 | (0.49 | - | 0.58) |  |  |
| Changes in sleep duration^3^ | 1.0 |  | 1.24 | (1.18 | - | 1.31) |  | 1.49 | (1.35 | - | 1.66) |  | 1.42 | (1.24 | - | 1.63) |  | 1.0 |  | 1.09 | (1.04 | - | 1.15) |  | 1.05 | (0.97 | - | 1.15) |  | 0.94 | (0.86 | - | 1.04) |  |  |
| Increased in consuming instant meals/soda | 1.0 |  | 0.91 | (0.86 | - | 0.96) |  | 0.68 | (0.58 | - | 0.80) |  | 0.63 | (0.50 | - | 0.80) |  | 1.0 |  | 0.81 | (0.77 | - | 0.86) |  | 0.45 | (0.38 | - | 0.52) |  | 0.31 | (0.25 | - | 0.38) |  |  |
| Increased in consuming delivery food | 1.0 |  | 0.72 | (0.69 | - | 0.76) |  | 0.50 | (0.43 | - | 0.57) |  | 0.34 | (0.27 | - | 0.42) |  | 1.0 |  | 0.76 | (0.73 | - | 0.80) |  | 0.37 | (0.33 | - | 0.42) |  | 0.25 | (0.21 | - | 0.29) |  |  |
| Increased in alcohol drinking^4^ | 1.0 |  | 1.17 | (1.06 | - | 1.28) |  | 1.11 | (0.92 | - | 1.35) |  | 1.14 | (0.87 | - | 1.48) |  | 1.0 |  | 0.87 | (0.78 | - | 0.97) |  | 0.63 | (0.48 | - | 0.81) |  | 0.49 | (0.34 | - | 0.70) |  |  |
| Increased in smoking amount^5^ | 1.0 |  | 1.32 | (1.19 | - | 1.46) |  | 1.49 | (1.23 | - | 1.80) |  | 1.47 | (1.14 | - | 1.88) |  | 1.0 |  | 1.19 | (0.87 | - | 1.63) |  | 1.22 | (0.76 | - | 1.95) |  | 1.62 | (1.00 | - | 2.65) |  |  |
| Abbreviations: OR, odds ratio; 95% CI, 95% confidence interval 1. adjusted for quarantine/isolation experience due to COVID-19 infection and recent experience of fever/coughing 2. adjusted for moderate physical activity (yes/no)  3. adjusted for sleep duration  4. adjusted for alcohol drinking frequencies  5. adjusted for smoking status (current/past) | | | | | | | | | | | | | | | | | | | | | | | | | | | | | | | | | | | |
