# Supplementary material for: Socioeconomic inequality in compliance with precautions and health behavior changes during the COVID-19 outbreak: an analysis of the Korean Community Health Survey 2020
Source: Epidemiol Health. 2022 Jan 9;44:e2022013. doi: 10.4178/epih.e2022013 (PMC8989472; doi:10.4178/epih.e2022013)
Supplement: Supplementary Material 3. — Odds ratio by sex for failure to comply with safety precautions and health behavior deterioration during COVID-19 outbreak according to education attainment in participants aged 65 or more [file epih-44-e2022013-suppl3.docx]

| Supplementary Material 3. Odds ratio by sex for failure to comply with safety precautions and health behavior deterioration during COVID-19 outbreak according to education attainment in participants aged 65 or more | | | | | | | | | | | | | | | | | | | | | | | | | | | | | | | | | | |
| --- | --- | --- | --- | --- | --- | --- | --- | --- | --- | --- | --- | --- | --- | --- | --- | --- | --- | --- | --- | --- | --- | --- | --- | --- | --- | --- | --- | --- | --- | --- | --- | --- | --- | --- |
| COVID19-related questionnaires | Men, age ≥ 65 (n=30,277) | | | | | | | | | | | | | | | |  | Women, age ≥ 65 (n=42,437) | | | | | | | | | | | | | | | | |
|  | College + |  | High school | | | |  | Middle school | | | |  | Elementary or less | | | |  | College + |  | High school | | | |  | Middle school | | | |  | Elementary or less | | | |  |
| Failure to comply with safety precautions^1^ |  |  |  |  |  |  |  |  |  |  |  |  |  |  |  |  |  |  |  |  |  |  |  |  |  |  |  |  |  |  |  |  |  |  |
| Not covering mouth while coughing | 1.0 |  | 1.48 | (1.18 | - | 1.85) |  | 1.76 | (1.39 | - | 2.22) |  | 2.40 | (1.94 | - | 2.96) |  | 1.0 |  | 1.24 | (0.80 | - | 1.92) |  | 1.57 | (1.03 | - | 2.41) |  | 2.55 | (1.72 | - | 3.79) |  |
| No regular ventilation | 1.0 |  | 1.53 | (0.95 | - | 2.46) |  | 2.35 | (1.45 | - | 3.82) |  | 2.60 | (1.66 | - | 4.06) |  | 1.0 |  | 1.41 | (0.78 | - | 2.53) |  | 1.05 | (0.53 | - | 2.07) |  | 1.71 | (0.99 | - | 2.98) |  |
| No regular disinfection | 1.0 |  | 1.07 | (0.97 | - | 1.18) |  | 1.17 | (1.05 | - | 1.30) |  | 1.56 | (1.41 | - | 1.72) |  | 1.0 |  | 1.26 | (1.09 | - | 1.46) |  | 1.44 | (1.24 | - | 1.66) |  | 1.87 | (1.63 | - | 2.14) |  |
| No mask wearing in indoor facilities | 1.0 |  | 1.24 | (0.61 | - | 2.50) |  | 1.19 | (0.59 | - | 2.41) |  | 2.24 | (1.18 | - | 4.24) |  | 1.0 |  | 0.64 | (0.19 | - | 2.11) |  | 1.14 | (0.40 | - | 3.30) |  | 1.33 | (0.52 | - | 3.42) |  |
| No mask wearing when hard to keep distance | 1.0 |  | 1.19 | (0.72 | - | 1.97) |  | 1.79 | (1.11 | - | 2.89) |  | 2.59 | (1.67 | - | 4.03) |  | 1.0 |  | 0.47 | (0.16 | - | 1.40) |  | 0.92 | (0.31 | - | 2.69) |  | 2.31 | (0.88 | - | 6.04) |  |
| Not keeping minimal physical distance | 1.0 |  | 1.41 | (1.04 | - | 1.93) |  | 1.42 | (1.03 | - | 1.96) |  | 1.78 | (1.32 | - | 2.41) |  | 1.0 |  | 1.25 | (0.73 | - | 2.15) |  | 1.65 | (0.94 | - | 2.91) |  | 2.17 | (1.27 | - | 3.71) |  |
| Not refrain from visiting hospitalized patients | 1.0 |  | 0.83 | (0.49 | - | 1.40) |  | 1.06 | (0.61 | - | 1.83) |  | 1.20 | (0.73 | - | 1.97) |  | 1.0 |  | 1.65 | (0.62 | - | 4.41) |  | 1.70 | (0.63 | - | 4.60) |  | 1.70 | (0.67 | - | 4.33) |  |
| Not refrain from going out | 1.0 |  | 0.76 | (0.55 | - | 1.06) |  | 0.68 | (0.48 | - | 0.96) |  | 1.03 | (0.74 | - | 1.42) |  | 1.0 |  | 0.94 | (0.49 | - | 1.80) |  | 1.06 | (0.58 | - | 1.95) |  | 1.22 | (0.69 | - | 2.14) |  |
| Health behavior deterioration |  |  |  |  |  |  |  |  |  |  |  |  |  |  |  |  |  |  |  |  |  |  |  |  |  |  |  |  |  |  |  |  |  |  |
| Decreased in physical activity^2^ | 1.0 |  | 0.92 | (0.82 | - | 1.02) |  | 0.89 | (0.79 | - | 1.00) |  | 0.73 | (0.65 | - | 0.81) |  | 1.0 |  | 1.06 | (0.90 | - | 1.25) |  | 0.92 | (0.79 | - | 1.08) |  | 0.75 | (0.65 | - | 0.87) |  |
| Changes in sleep duration^3^ | 1.0 |  | 0.93 | (0.81 | - | 1.07) |  | 1.01 | (0.87 | - | 1.16) |  | 0.91 | (0.79 | - | 1.04) |  | 1.0 |  | 1.09 | (1.04 | - | 1.15) |  | 1.05 | (0.97 | - | 1.15) |  | 0.94 | (0.86 | - | 1.04) |  |
| Increased in consuming instant meals/soda | 1.0 |  | 0.55 | (0.41 | - | 0.74) |  | 0.48 | (0.34 | - | 0.66) |  | 0.49 | (0.37 | - | 0.66) |  | 1.0 |  | 0.61 | (0.42 | - | 0.89) |  | 0.36 | (0.24 | - | 0.53) |  | 0.28 | (0.19 | - | 0.39) |  |
| Increased in consuming delivery food | 1.0 |  | 0.54 | (0.43 | - | 0.68) |  | 0.38 | (0.29 | - | 0.49) |  | 0.27 | (0.21 | - | 0.36) |  | 1.0 |  | 0.64 | (0.46 | - | 0.88) |  | 0.38 | (0.27 | - | 0.53) |  | 0.29 | (0.21 | - | 0.39) |  |
| Increased in alcohol drinking^4^ | 1.0 |  | 0.71 | (0.48 | - | 1.07) |  | 0.76 | (0.51 | - | 1.14) |  | 0.73 | (0.49 | - | 1.10) |  | 1.0 |  | 0.55 | (0.17 | - | 1.77) |  | 0.80 | (0.30 | - | 2.17) |  | 1.27 | (0.49 | - | 3.24) |  |
| Increased in smoking amount^5^ | 1.0 |  | 1.22 | (0.70 |  | 2.14) |  | 0.48 | (0.25 |  | 0.93) |  | 1.08 | (0.63 |  | 1.86) |  | 1.0 |  | n/a |  |  |  |  | n/a |  |  |  |  | n/a |  |  |  |  |
| Values are presented as odds ratio (95% confidence interval). 1. adjusted for quarantine/isolation experience due to COVID-19 infection and recent experience of fever/coughing 2. adjusted for moderate physical activity (yes/no)  3. adjusted for sleep duration  4. adjusted for alcohol drinking frequencies  5. adjusted for smoking status (current/past) | | | | | | | | | | | | | | | | | | | | | | | | | | | | | | | | | | |
