# Supplementary material for: Socioeconomic inequality in compliance with precautions and health behavior changes during the COVID-19 outbreak: an analysis of the Korean Community Health Survey 2020
Source: Epidemiol Health. 2022 Jan 9;44:e2022013. doi: 10.4178/epih.e2022013 (PMC8989472; doi:10.4178/epih.e2022013)
Supplement: Supplementary Material 4. — Odds ratio by sex for failure to comply with safety precautions and health behavior deterioration during COVID-19 outbreak according to education attainment in participants aged under 65 [file epih-44-e2022013-suppl4.docx]

| Supplementary Material 4. Odds ratio by sex for failure to comply with safety precautions and health behavior deterioration during COVID-19 outbreak according to education attainment in participants aged under 65 | | | | | | | | | | | | | | | | | | | | | | | | | | | | | | | | | | |
| --- | --- | --- | --- | --- | --- | --- | --- | --- | --- | --- | --- | --- | --- | --- | --- | --- | --- | --- | --- | --- | --- | --- | --- | --- | --- | --- | --- | --- | --- | --- | --- | --- | --- | --- |
| COVID19-related questionnaires | Men, age < 65 (n=64,929) | | | | | | | | | | | | | | | |  | Women, age < 65 (n=73,025) | | | | | | | | | | | | | | | | |
|  | Q4  (highest) |  | Q3 | | | |  | Q2 | | | |  | Q1 (lowest) | | | |  | Q4  (highest) |  | Q3 | | | |  | Q2 | | | |  | Q1 (lowest) | | | |  |
|  | OR |  | OR | 95% CI | | |  | OR | 95% CI | | |  | OR | 95% CI | | |  | OR |  | OR | 95% CI | | |  | OR | 95% CI | | |  | OR | 95% CI | | |  |
| Failure to comply with safety precautions^1^ |  |  |  |  |  |  |  |  |  |  |  |  |  |  |  |  |  |  |  |  |  |  |  |  |  |  |  |  |  |  |  |  |  |  |
| Not covering mouth while coughing | 1.0 |  | 1.16 | (1.02 | - | 1.33) |  | 1.31 | (1.14 | - | 1.50) |  | 1.56 | (1.34 | - | 1.81) |  | 1.0 |  | 1.16 | (0.98 | - | 1.37) |  | 1.62 | (1.38 | - | 1.90) |  | 1.75 | (1.47 | - | 2.07) |  |
| No regular ventilation | 1.0 |  | 1.01 | (0.86 | - | 1.18) |  | 0.88 | (0.74 | - | 1.04) |  | 1.01 | (0.83 | - | 1.22) |  | 1.0 |  | 1.00 | (0.81 | - | 1.24) |  | 0.97 | (0.79 | - | 1.21) |  | 1.17 | (0.91 | - | 1.50) |  |
| No regular disinfection | 1.0 |  | 1.08 | (1.03 | - | 1.14) |  | 1.23 | (1.17 | - | 1.30) |  | 1.78 | (1.67 | - | 1.90) |  | 1.0 |  | 1.05 | (1.00 | - | 1.11) |  | 1.16 | (1.10 | - | 1.22) |  | 1.34 | (1.26 | - | 1.43) |  |
| No mask wearing in indoor facilities | 1.0 |  | 2.01 | (1.44 | - | 2.82) |  | 1.52 | (1.04 | - | 2.22) |  | 2.53 | (1.72 | - | 3.73) |  | 1.0 |  | 0.84 | (0.49 | - | 1.44) |  | 1.00 | (0.59 | - | 1.70) |  | 1.61 | (0.90 | - | 2.87) |  |
| No mask wearing when hard to keep distance | 1.0 |  | 1.14 | (0.90 | - | 1.44) |  | 0.97 | (0.77 | - | 1.23) |  | 1.44 | (1.09 | - | 1.90) |  | 1.0 |  | 1.05 | (0.69 | - | 1.59) |  | 1.18 | (0.81 | - | 1.72) |  | 1.39 | (0.94 | - | 2.04) |  |
| Not keeping minimal physical distance | 1.0 |  | 1.06 | (0.95 | - | 1.19) |  | 1.01 | (0.90 | - | 1.14) |  | 1.01 | (0.88 | - | 1.17) |  | 1.0 |  | 1.04 | (0.92 | - | 1.18) |  | 1.08 | (0.95 | - | 1.23) |  | 0.98 | (0.84 | - | 1.15) |  |
| Not refrain from visiting hospitalized patients | 1.0 |  | 1.15 | (0.87 | - | 1.52) |  | 1.25 | (0.93 | - | 1.67) |  | 1.79 | (1.30 | - | 2.46) |  | 1.0 |  | 1.04 | (0.79 | - | 1.36) |  | 1.22 | (0.93 | - | 1.60) |  | 1.26 | (0.95 | - | 1.67) |  |
| Not refrain from going out | 1.0 |  | 1.10 | (0.94 | - | 1.27) |  | 1.08 | (0.92 | - | 1.27) |  | 1.04 | (0.86 | - | 1.26) |  | 1.0 |  | 0.98 | (0.83 | - | 1.17) |  | 0.94 | (0.78 | - | 1.13) |  | 1.16 | (0.94 | - | 1.43) |  |
| Health behavior deterioration |  |  |  |  |  |  |  |  |  |  |  |  |  |  |  |  |  |  |  |  |  |  |  |  |  |  |  |  |  |  |  |  |  |  |
| Decreased in physical activity^2^ | 1.0 |  | 0.88 | (0.83 | - | 0.93) |  | 0.83 | (0.79 | - | 0.88) |  | 0.81 | (0.76 | - | 0.87) |  | 1.0 |  | 0.85 | (0.80 | - | 0.90) |  | 0.81 | (0.77 | - | 0.86) |  | 0.78 | (0.73 | - | 0.83) |  |
| Changes in sleep duration^3^ | 1.0 |  | 1.02 | (0.96 | - | 1.09) |  | 1.19 | (1.11 | - | 1.27) |  | 1.37 | (1.27 | - | 1.48) |  | 1.0 |  | 1.00 | (0.94 | - | 1.06) |  | 1.12 | (1.05 | - | 1.18) |  | 1.26 | (1.18 | - | 1.35) |  |
| Increased in consuming instant meals/soda | 1.0 |  | 0.93 | (0.87 | - | 1.00) |  | 1.02 | (0.95 | - | 1.11) |  | 0.83 | (0.77 | - | 0.90) |  | 1.0 |  | 0.84 | (0.78 | - | 0.89) |  | 0.76 | (0.71 | - | 0.82) |  | 0.67 | (0.62 | - | 0.73) |  |
| Increased in consuming delivery food | 1.0 |  | 0.79 | (0.75 | - | 0.84) |  | 0.71 | (0.67 | - | 0.76) |  | 0.50 | (0.47 | - | 0.54) |  | 1.0 |  | 0.80 | (0.75 | - | 0.85) |  | 0.68 | (0.64 | - | 0.73) |  | 0.58 | (0.54 | - | 0.62) |  |
| Increased in alcohol drinking^4^ | 1.0 |  | 1.03 | (0.92 | - | 1.15) |  | 1.14 | (1.02 | - | 1.28) |  | 1.27 | (1.10 | - | 1.46) |  | 1.0 |  | 0.91 | (0.81 | - | 1.02) |  | 0.79 | (0.69 | - | 0.90) |  | 0.75 | (0.63 | - | 0.89) |  |
| Increased in smoking amount^5^ | 1.0 |  | 1.12 | (0.98 | - | 1.27) |  | 1.30 | (1.14 | - | 1.48) |  | 1.53 | (1.31 | - | 1.78) |  | 1.0 |  | 1.05 | (0.70 | - | 1.58) |  | 0.96 | (0.66 | - | 1.41) |  | 1.45 | (0.97 | - | 2.16) |  |
| Abbreviations: OR, odds ratio; 95% CI, 95% confidence interval 1. adjusted for quarantine/isolation experience due to COVID-19 infection and recent experience of fever/coughing 2. adjusted for moderate physical activity (yes/no)  3. adjusted for sleep duration  4. adjusted for alcohol drinking frequencies  5. adjusted for smoking status (current/past) | | | | | | | | | | | | | | | | | | | | | | | | | | | | | | | | | | |
