# Supplementary material for: Socioeconomic inequality in compliance with precautions and health behavior changes during the COVID-19 outbreak: an analysis of the Korean Community Health Survey 2020
Source: Epidemiol Health. 2022 Jan 9;44:e2022013. doi: 10.4178/epih.e2022013 (PMC8989472; doi:10.4178/epih.e2022013)
Supplement: Supplementary Material 5. — Odds ratio by sex for failure to comply with safety precautions and health behavior deterioration during COVID-19 outbreak according to education attainment in participants aged 65 or more [file epih-44-e2022013-suppl5.docx]

| Supplementary Material 5. Odds ratio by sex for failure to comply with safety precautions and health behavior deterioration during COVID-19 outbreak according to education attainment in participants aged 65 or more | | | | | | | | | | | | | | | | | | | | | | | | | | | | | | | | | | |
| --- | --- | --- | --- | --- | --- | --- | --- | --- | --- | --- | --- | --- | --- | --- | --- | --- | --- | --- | --- | --- | --- | --- | --- | --- | --- | --- | --- | --- | --- | --- | --- | --- | --- | --- |
| COVID19-related questionnaires | Men, age ≥ 65 (n=29,803) | | | | | | | | | | | | | | | |  | Women, age ≥ 65 (n=41,698) | | | | | | | | | | | | | | | | |
|  | Q4 (highest) |  | Q3 | | | |  | Q2 | | | |  | Q1 (lowest) | | | |  | Q4 (highest) |  | Q3 | | | |  | Q2 | | | |  | Q1 (lowest) | | | |  |
|  | OR |  | OR | 95% CI | | |  | OR | 95% CI | | |  | OR | 95% CI | | |  | OR |  | OR | 95% CI | | |  | OR | 95% CI | | |  | OR | 95% CI | | |  |
| Failure to comply with safety precautions^1^ |  |  |  |  |  |  |  |  |  |  |  |  |  |  |  |  |  |  |  |  |  |  |  |  |  |  |  |  |  |  |  |  |  |  |
| Not covering mouth while coughing | 1.0 |  | 1.01 | (0.71 | - | 1.44) |  | 1.08 | (0.79 | - | 1.48) |  | 1.45 | (1.08 | - | 1.95) |  | 1.0 |  | 0.96 | (0.66 | - | 1.39) |  | 0.99 | (0.72 | - | 1.38) |  | 1.50 | (1.11 | - | 2.02) |  |
| No regular ventilation | 1.0 |  | 1.66 | (0.84 | - | 3.30) |  | 1.63 | (0.86 | - | 3.07) |  | 1.90 | (1.04 | - | 3.47) |  | 1.0 |  | 1.83 | (0.88 | - | 3.77) |  | 1.23 | (0.63 | - | 2.41) |  | 1.63 | (0.86 | - | 3.11) |  |
| No regular disinfection | 1.0 |  | 1.20 | (1.02 | - | 1.41) |  | 1.26 | (1.09 | - | 1.46) |  | 1.90 | (1.65 | - | 2.19) |  | 1.0 |  | 0.88 | (0.75 | - | 1.03) |  | 1.04 | (0.90 | - | 1.20) |  | 1.49 | (1.31 | - | 1.71) |  |
| No mask wearing in indoor facilities | 1.0 |  | 2.07 | (0.70 | - | 6.10) |  | 1.65 | (0.63 | - | 4.34) |  | 2.02 | (0.81 | - | 5.07) |  | 1.0 |  | 1.21 | (0.32 | - | 4.62) |  | 1.08 | (0.32 | - | 3.70) |  | 1.45 | (0.46 | - | 4.61) |  |
| No mask wearing when hard to keep distance | 1.0 |  | 1.38 | (0.67 | - | 2.87) |  | 1.58 | (0.82 | - | 3.05) |  | 1.93 | (1.03 | - | 3.60) |  | 1.0 |  | 1.96 | (0.91 | - | 4.20) |  | 2.51 | (1.30 | - | 4.88) |  | 3.89 | (2.10 | - | 7.21) |  |
| Not keeping minimal physical distance | 1.0 |  | 1.14 | (0.70 | - | 1.86) |  | 1.25 | (0.81 | - | 1.93) |  | 1.29 | (0.84 | - | 1.98) |  | 1.0 |  | 1.01 | (0.61 | - | 1.69) |  | 1.17 | (0.75 | - | 1.82) |  | 1.37 | (0.88 | - | 2.12) |  |
| Not refrain from visiting hospitalized patients | 1.0 |  | 1.20 | (0.56 | - | 2.57) |  | 0.92 | (0.44 | - | 1.90) |  | 1.12 | (0.56 | - | 2.25) |  | 1.0 |  | 2.20 | (0.79 | - | 6.12) |  | 2.33 | (0.88 | - | 6.18) |  | 2.71 | (1.04 | - | 7.06) |  |
| Not refrain from going out | 1.0 |  | 0.72 | (0.42 | - | 1.23) |  | 0.82 | (0.52 | - | 1.29) |  | 0.78 | (0.51 | - | 1.19) |  | 1.0 |  | 0.95 | (0.54 | - | 1.68) |  | 0.94 | (0.58 | - | 1.55) |  | 1.06 | (0.68 | - | 1.68) |  |
| Health behavior deterioration |  |  |  |  |  |  |  |  |  |  |  |  |  |  |  |  |  |  |  |  |  |  |  |  |  |  |  |  |  |  |  |  |  |  |
| Decreased in physical activity^2^ | 1.0 |  | 0.90 | (0.75 | - | 1.07) |  | 0.87 | (0.74 | - | 1.02) |  | 0.86 | (0.74 | - | 1.01) |  | 1.0 |  | 0.95 | (0.80 | - | 1.13) |  | 0.87 | (0.75 | - | 1.02) |  | 0.82 | (0.70 | - | 0.94) |  |
| Changes in sleep duration^3^ | 1.0 |  | 1.03 | (0.82 | - | 1.30) |  | 1.00 | (0.81 | - | 1.23) |  | 1.28 | (1.05 | - | 1.56) |  | 1.0 |  | 0.91 | (0.73 | - | 1.15) |  | 1.08 | (0.89 | - | 1.32) |  | 1.10 | (0.91 | - | 1.33) |  |
| Increased in consuming instant meals/soda | 1.0 |  | 0.63 | (0.49 | - | 0.82) |  | 0.75 | (0.55 | - | 1.02) |  | 0.70 | (0.50 | - | 0.97) |  | 1.0 |  | 0.91 | (0.69 | - | 1.19) |  | 0.87 | (0.65 | - | 1.15) |  | 0.83 | (0.58 | - | 1.19) |  |
| Increased in consuming delivery food | 1.0 |  | 0.52 | (0.42 | - | 0.64) |  | 0.48 | (0.36 | - | 0.63) |  | 0.32 | (0.23 | - | 0.43) |  | 1.0 |  | 0.61 | (0.49 | - | 0.75) |  | 0.47 | (0.36 | - | 0.60) |  | 0.52 | (0.38 | - | 0.70) |  |
| Increased in alcohol drinking^4^ | 1.0 |  | 0.97 | (0.52 | - | 1.81) |  | 0.88 | (0.50 | - | 1.54) |  | 0.81 | (0.47 | - | 1.40) |  | 1.0 |  | 1.38 | (0.36 | - | 5.32) |  | 1.05 | (0.31 | - | 3.56) |  | 1.33 | (0.41 | - | 4.32) |  |
| Increased in smoking amount^5^ | 1.0 |  | 2.33 | (1.04 |  | 5.20) |  | 1.03 | (0.48 |  | 2.20) |  | 1.29 | (0.62 |  | 2.69) |  | 1.0 |  | n/a |  |  |  |  | n/a |  |  |  |  | n/a |  |  |  |  |
| Abbreviations: OR, odds ratio; 95% CI, 95% confidence interval 1. adjusted for quarantine/isolation experience due to COVID-19 infection and recent experience of fever/coughing 2. adjusted for moderate physical activity (yes/no)  3. adjusted for sleep duration  4. adjusted for alcohol drinking frequencies  5. adjusted for smoking status (current/past) | | | | | | | | | | | | | | | | | | | | | | | | | | | | | | | | | | |
