# Supplementary material for: Socioeconomic inequality in compliance with precautions and health behavior changes during the COVID-19 outbreak: an analysis of the Korean Community Health Survey 2020
Source: Epidemiol Health. 2022 Jan 9;44:e2022013. doi: 10.4178/epih.e2022013 (PMC8989472; doi:10.4178/epih.e2022013)
Supplement: Supplementary Material 6. — Estimates of relative index of inequality (RII) assessed by educational attainment failure to comply with precautions and health behavior changes by sex and age [file epih-44-e2022013-suppl6.docx]

| Supplementary Material 6. Estimates of relative index of inequality (RII) assessed by educational attainment failure to comply with precautions and health behavior changes by sex and age | | | | | | | | | | | | | | | | | | | | | | | | | |
| --- | --- | --- | --- | --- | --- | --- | --- | --- | --- | --- | --- | --- | --- | --- | --- | --- | --- | --- | --- | --- | --- | --- | --- | --- | --- |
| COVID19-related questionnaires | Entire population | | | |  | Men, < aged 65 | | | |  | Men, ≥ aged 65 | | | |  | Women, < aged 65 | | | |  | Women, ≥ aged 65 | | | | |
|  | RII | 95%CI | | |  | RII | 95%CI | | |  | RII | 95%CI | | |  | RII | 95%CI | | |  | RII | 95%CI | | |  |
| Failure to comply with safety precautions^1^ |  |  |  |  |  |  |  |  |  |  |  |  |  |  |  |  |  |  |  |  |  |  |  |  |  |
| Not covering mouth while coughing | 3.05 | (2.65 | - | 3.52) |  | 2.75 | (2.26 | - | 3.35) |  | 2.89 | (2.32 | - | 3.60) |  | 3.46 | (2.66 | - | 4.51) |  | 3.63 | (2.76 | - | 4.78) |  |
| No mask wearing when hard to keep distance | 2.79 | (2.12 | - | 3.67) |  | 3.02 | (2.09 | - | 4.37) |  | 3.91 | (2.68 | - | 5.71) |  | 2.19 | (1.27 | - | 3.79) |  | 9.37 | (4.54 | - | 19.35) |  |
| No mask wearing in indoor facilities | 2.76 | (1.83 | - | 4.16) |  | 4.05 | (2.40 | - | 6.84) |  | 3.07 | (1.63 | - | 5.78) |  | 1.07 | (0.50 | - | 2.33) |  | 2.20 | (0.88 | - | 5.48) |  |
| No regular ventilation | 2.20 | (1.78 | - | 2.71) |  | 1.64 | (1.26 | - | 2.14) |  | 3.07 | (2.07 | - | 4.57) |  | 1.96 | (1.42 | - | 2.72) |  | 2.03 | (1.17 | - | 3.53) |  |
| No regular disinfection | 2.05 | (1.93 | - | 2.18) |  | 1.90 | (1.75 | - | 2.05) |  | 1.87 | (1.68 | - | 2.08) |  | 1.49 | (1.37 | - | 1.62) |  | 2.13 | (1.90 | - | 2.39) |  |
| Not keeping minimal physical distancing | 1.63 | (1.41 | - | 1.88) |  | 1.44 | (1.21 | - | 1.72) |  | 1.87 | (1.39 | - | 2.52) |  | 1.61 | (1.30 | - | 1.98) |  | 2.54 | (1.77 | - | 3.64) |  |
| Not refrain from going out | 1.50 | (1.22 | - | 1.84) |  | 1.46 | (1.14 | - | 1.87) |  | 1.20 | (0.80 | - | 1.81) |  | 1.31 | (0.97 | - | 1.76) |  | 1.48 | (0.96 | - | 2.29) |  |
| Not refrain from visiting patients in hospital | 1.50 | (1.13 | - | 1.99) |  | 1.75 | (1.19 | - | 2.59) |  | 1.51 | (0.87 | - | 2.61) |  | 1.35 | (0.88 | - | 2.07) |  | 1.20 | (0.68 | - | 2.12) |  |
| Health behavior deterioration |  |  |  |  |  |  |  |  |  |  |  |  |  |  |  |  |  |  |  |  |  |  |  |  |  |
| Increased in smoking amount^2^ | 2.10 | (1.71 | - | 2.59) |  | 2.06 | (1.70 | - | 2.50) |  | 1.04 | (0.54 | - | 2.01) |  | 3.35 | (1.95 | - | 5.76) |  | 3.82 | (0.81 | - | 18.08) |  |
| Changes in sleep duration^3^ | 1.21 | (1.13 | - | 1.30) |  | 1.69 | (1.53 | - | 1.87) |  | 0.93 | (0.80 | - | 1.09) |  | 1.11 | (1.01 | - | 1.22) |  | 0.73 | (0.63 | - | 0.85) |  |
| Increased in alcohol drinking^4^ | 0.99 | (0.85 | - | 1.16) |  | 1.73 | (1.46 | - | 2.05) |  | 1.02 | (0.65 | - | 1.62) |  | 0.73 | (0.58 | - | 0.91) |  | 2.72 | (0.94 | - | 7.86) |  |
| Decreased in physical activity^5^ | 0.59 | (0.55 | - | 0.62) |  | 0.60 | (0.56 | - | 0.66) |  | 0.67 | (0.59 | - | 0.75) |  | 0.48 | (0.44 | - | 0.53) |  | 0.58 | (0.52 | - | 0.66) |  |
| Increased in consuming instant meals/soda | 0.42 | (0.38 | - | 0.46) |  | 0.73 | (0.65 | - | 0.82) |  | 0.45 | (0.29 | - | 0.69) |  | 0.45 | (0.40 | - | 0.51) |  | 0.21 | (0.13 | - | 0.34) |  |
| Increased in consuming delivery food | 0.27 | (0.24 | - | 0.29) |  | 0.43 | (0.39 | - | 0.47) |  | 0.18 | (0.12 | - | 0.26) |  | 0.36 | (0.33 | - | 0.40) |  | 0.23 | (0.16 | - | 0.32) |  |
| Abbreviations: OR, odds ratio; 95% CI, 95% confidence interval 1. adjusted for quarantine/isolation experience due to COVID-19 infection and recent experience of fever/coughing 2. adjusted for smoking status (current/past)  3. adjusted for sleep duration  4. adjusted for alcohol drinking frequencies  5. adjusted for moderate physical activity (yes/no) | | | | | | | | | | | | | | | | | | | | | | | | | |
