# Supplementary material for: Socioeconomic inequality in compliance with precautions and health behavior changes during the COVID-19 outbreak: an analysis of the Korean Community Health Survey 2020
Source: Epidemiol Health. 2022 Jan 9;44:e2022013. doi: 10.4178/epih.e2022013 (PMC8989472; doi:10.4178/epih.e2022013)
Supplement: Supplementary Material 7. — Estimates of relative index of inequality (RII) assessed by household income for failure to comply with precautions and health behavior deterioration by sex and age [file epih-44-e2022013-suppl7.docx]

| Supplementary Material 7. Estimates of relative index of inequality (RII) assessed by household income for failure to comply with precautions and health behavior deterioration by sex and age | | | | | | | | | | | | | | | | | | | | | | | | | |
| --- | --- | --- | --- | --- | --- | --- | --- | --- | --- | --- | --- | --- | --- | --- | --- | --- | --- | --- | --- | --- | --- | --- | --- | --- | --- |
| COVID19-related questionnaires | Entire population | | | |  | Men, < aged 65 | | | |  | Men, ≥ aged 65 | | | |  | Women, < aged 65 | | | |  | Women, ≥ aged 65 | | | |  |
|  | RII | 95%CI | | |  | RII | 95%CI | | |  | RII | 95%CI | | |  | RII | 95%CI | | |  | RII | 95%CI | | |  |
| Failure to comply with safety precautions |  |  |  |  |  |  |  |  |  |  |  |  |  |  |  |  |  |  |  |  |  |  |  |  |  |
| No mask wearing in indoor facilities* | 1.97 | (1.37 | - | 2.85) |  | 2.56 | (1.65 | - | 3.95) |  | 1.01 | (0.51 | - | 2.00) |  | 1.27 | (0.62 | - | 2.59) |  | 2.22 | (1.15 | - | 4.27) |  |
| No regular disinfection | 1.96 | (1.86 | - | 2.07) |  | 1.79 | (1.66 | - | 1.93) |  | 2.55 | (2.26 | - | 2.88) |  | 1.39 | (1.29 | - | 1.50) |  | 2.24 | (2.02 | - | 2.47) |  |
| Not covering mouth while coughing* | 1.93 | (1.70 | - | 2.18) |  | 1.73 | (1.45 | - | 2.06) |  | 1.96 | (1.57 | - | 2.45) |  | 2.14 | (1.75 | - | 2.61) |  | 2.19 | (1.81 | - | 2.65) |  |
| Not refrain from visiting hospitalized patients* | 1.59 | (1.22 | - | 2.08) |  | 1.67 | (1.14 | - | 2.46) |  | 1.45 | (0.73 | - | 2.88) |  | 1.49 | (1.05 | - | 2.12) |  | 1.58 | (0.95 | - | 2.64) |  |
| No mask wearing when hard to keep distance* | 1.44 | (1.13 | - | 1.85) |  | 1.25 | (0.90 | - | 1.73) |  | 1.45 | (1.00 | - | 2.11) |  | 1.29 | (0.80 | - | 2.07) |  | 3.77 | (2.65 | - | 5.38) |  |
| Not refrain from going out* | 1.20 | (1.02 | - | 1.42) |  | 1.04 | (0.84 | - | 1.29) |  | 0.97 | (0.65 | - | 1.45) |  | 1.05 | (0.82 | - | 1.35) |  | 1.28 | (0.90 | - | 1.84) |  |
| No regular ventilation | 1.20 | (1.01 | - | 1.43) |  | 0.98 | (0.79 | - | 1.23) |  | 1.82 | (1.24 | - | 2.66) |  | 1.14 | (0.85 | - | 1.54) |  | 1.21 | (0.85 | - | 1.72) |  |
| Not keeping minimal physical distance* | 1.06 | (0.94 | - | 1.20) |  | 0.99 | (0.84 | - | 1.16) |  | 1.58 | (1.13 | - | 2.21) |  | 1.01 | (0.85 | - | 1.21) |  | 1.56 | (1.19 | - | 2.05) |  |
| Health behavior deterioration |  |  |  |  |  |  |  |  |  |  |  |  |  |  |  |  |  |  |  |  |  |  |  |  |  |
| Increased in smoking amount | 1.67 | (1.40 | - | 2.00) |  | 1.71 | (1.43 | - | 2.04) |  | 1.03 | (0.54 | - | 1.97) |  | 2.24 | (1.36 | - | 3.67) |  | 1.37 | (0.45 | - | 4.21) |  |
| Changes in sleep duration | 1.36 | (1.28 | - | 1.45) |  | 1.46 | (1.33 | - | 1.60) |  | 1.51 | (1.27 | - | 1.79) |  | 1.32 | (1.22 | - | 1.43) |  | 1.30 | (1.13 | - | 1.49) |  |
| Increased in alcohol drinking | 0.86 | (0.76 | - | 0.98) |  | 1.31 | (1.12 | - | 1.53) |  | 0.94 | (0.59 | - | 1.49) |  | 0.68 | (0.57 | - | 0.81) |  | 1.56 | (0.63 | - | 3.86) |  |
| Decreased in physical activity | 0.77 | (0.73 | - | 0.81) |  | 0.75 | (0.70 | - | 0.81) |  | 0.91 | (0.80 | - | 1.03) |  | 0.74 | (0.68 | - | 0.80) |  | 0.82 | (0.74 | - | 0.92) |  |
| Increased in consuming instant meals/soda | 0.61 | (0.56 | - | 0.66) |  | 0.83 | (0.75 | - | 0.92) |  | 0.72 | (0.45 | - | 1.16) |  | 0.58 | (0.53 | - | 0.64) |  | 0.73 | (0.46 | - | 1.15) |  |
| Increased in consuming delivery food | 0.37 | (0.35 | - | 0.40) |  | 0.43 | (0.40 | - | 0.47) |  | 0.21 | (0.14 | - | 0.33) |  | 0.48 | (0.44 | - | 0.52) |  | 0.28 | (0.19 | - | 0.42) |  |
| Abbreviations: OR, odds ratio; 95% CI, 95% confidence interval 1. adjusted for quarantine/isolation experience due to COVID-19 infection and recent experience of fever/coughing 2. adjusted for smoking status (current/past)  3. adjusted for sleep duration  4. adjusted for alcohol drinking frequencies  5. adjusted for moderate physical activity (yes/no) | | | | | | | | | | | | | | | | | | | | | | | | | |
