# Supplementary material for: Socioeconomic inequality in compliance with precautions and health behavior changes during the COVID-19 outbreak: an analysis of the Korean Community Health Survey 2020
Source: Epidemiol Health. 2022 Jan 9;44:e2022013. doi: 10.4178/epih.e2022013 (PMC8989472; doi:10.4178/epih.e2022013)
Supplement: Supplementary Material 8. — Age-standardized rates of COVID-19 safety precautions compliance and health behavior deterioration occurrence by educational attainment in men [file epih-44-e2022013-suppl8.docx]

| Supplementary Material 8. Age-standardized rates of COVID-19 safety precautions compliance and health behavior deterioration occurrence by educational attainment in men | | | | | | | | | | | | | | | | | | | |
| --- | --- | --- | --- | --- | --- | --- | --- | --- | --- | --- | --- | --- | --- | --- | --- | --- | --- | --- | --- |
| COVID19-related questionnaires | Education attainment | | | | | | | | | | | | | | | | | | |
|  | Elementary or less | | | |  | Middle school graduated | | | |  | High school graduated | | | |  | College or more | | | |
|  | rate, % | 95% CI | | |  | rate, % | 95% CI | | |  | rate, % | 95% CI | | |  | rate, % | 95% CI | | |
| Comply with safety precautions |  |  |  |  |  |  |  |  |  |  |  |  |  |  |  |  |  |  |  |
| Covering mouth while coughing* | 90.2 | (86.2 | - | 94.2) |  | 93.0 | (91.7 | - | 94.2) |  | 94.1 | (93.7 | - | 94.5) |  | 96.0 | (95.7 | - | 96.3) |
| Regular ventilation | 96.7 | (94.9 | - | 98.6) |  | 96.8 | (95.3 | - | 98.4) |  | 97.4 | (97.1 | - | 97.6) |  | 97.8 | (97.6 | - | 98.0) |
| Regular disinfection | 53.9 | (48.1 | - | 59.7) |  | 55.3 | (52.0 | - | 58.6) |  | 58.8 | (58.0 | - | 59.6) |  | 63.5 | (62.9 | - | 64.2) |
| Mask wearing in indoor facilities* | 97.7 | (95.8 | - | 99.7) |  | 99.0 | (98.6 | - | 99.4) |  | 99.4 | (99.3 | - | 99.5) |  | 99.6 | (99.5 | - | 99.7) |
| Mask wearing when hard keep distance* | 97.9 | (96.7 | - | 99.1) |  | 97.8 | (96.7 | - | 99.0) |  | 99.0 | (98.8 | - | 99.1) |  | 99.3 | (99.2 | - | 99.4) |
| Keeping minimal physical distance* | 96.7 | (95.0 | - | 98.4) |  | 94.1 | (92.3 | - | 95.8) |  | 95.0 | (94.7 | - | 95.4) |  | 95.6 | (95.4 | - | 95.9) |
| Refrain from visiting hospitalized patients* | 99.2 | (98.9 | - | 99.5) |  | 97.6 | (96.5 | - | 98.7) |  | 97.8 | (97.4 | - | 98.1) |  | 98.3 | (98.0 | - | 98.5) |
| Refrain from going out* | 95.7 | (92.5 | - | 99.0) |  | 97.0 | (95.9 | - | 98.1) |  | 96.8 | (96.5 | - | 97.1) |  | 97.0 | (96.7 | - | 97.2) |
| Health behavior deterioration |  |  |  |  |  |  |  |  |  |  |  |  |  |  |  |  |  |  |  |
| Decreased in physical activity† | 52.9 | (46.7 | - | 59.2) |  | 47.3 | (43.7 | - | 50.9) |  | 51.1 | (50.3 | - | 51.9) |  | 55.7 | (55.0 | - | 56.4) |
| Changes in sleep duration | 30.6 | (23.9 | - | 37.3) |  | 24.5 | (21.4 | - | 27.6) |  | 20.0 | (19.3 | - | 20.6) |  | 17.1 | (16.6 | - | 17.6) |
| Increased in consuming instant meals/soda† | 14.0 | (8.1 | - | 19.9) |  | 18.9 | (15.5 | - | 22.2) |  | 20.3 | (19.5 | - | 21.0) |  | 21.6 | (21.0 | - | 22.2) |
| Increased in consuming delivery food† | 24.1 | (17.4 | - | 30.9) |  | 33.1 | (29.1 | - | 37.0) |  | 36.7 | (35.9 | - | 37.6) |  | 43.8 | (43.0 | - | 44.5) |
| Increased in alcohol drinking† | 11.0 | (5.4 | - | 16.5) |  | 10.0 | (7.6 | - | 12.5) |  | 7.8 | (7.3 | - | 8.3) |  | 6.2 | (5.8 | - | 6.5) |
| Increased in smoking† | 11.5 | (7.0 | - | 16.0) |  | 12.1 | (9.3 | - | 14.9) |  | 9.9 | (9.3 | - | 10.6) |  | 7.3 | (6.8 | - | 7.8) |
| Abbreviations: 95% CI, 95% confidence interval *Excluded participants who responded as 'not applicable' during last 1 week †Excluded participants who responded as 'not applicable' | | | | | | | | | | | | | | | | | | | |
