# Supplementary material for: Socioeconomic inequality in compliance with precautions and health behavior changes during the COVID-19 outbreak: an analysis of the Korean Community Health Survey 2020
Source: Epidemiol Health. 2022 Jan 9;44:e2022013. doi: 10.4178/epih.e2022013 (PMC8989472; doi:10.4178/epih.e2022013)
Supplement: Supplementary Material 9. — Age-standardized rates of COVID-19 safety precautions compliance and health behavior deterioration occurrence by educational attainment in women [file epih-44-e2022013-suppl9.docx]

| Supplementary Material 9. Age-standardized rates of COVID-19 safety precautions compliance and health behavior deterioration occurrence by educational attainment in women. | | | | | | | | | | | | | | | | | | | |
| --- | --- | --- | --- | --- | --- | --- | --- | --- | --- | --- | --- | --- | --- | --- | --- | --- | --- | --- | --- |
| COVID19-related questionnaires | Education attainment | | | | | | | | | | | | | | | | | | |
|  | Elementary or less | | | |  | Middle school graduated | | | |  | High school graduated | | | |  | College or more | | | |
|  | rate, % | 95% CI | | |  | rate, % | 95% CI | | |  | rate, % | 95% CI | | |  | rate, % | 95% CI | | |
| Comply with safety precautions |  |  |  |  |  |  |  |  |  |  |  |  |  |  |  |  |  |  |  |
| Covering mouth while coughing* | 92.7 | (90.4 | - | 94.9) |  | 94.7 | (93.3 | - | 96.1) |  | 96.3 | (96.0 | - | 96.6) |  | 97.4 | (97.0 | - | 97.7) |
| Regular ventilation | 99.1 | (98.8 | - | 99.4) |  | 98.3 | (97.5 | - | 99.2) |  | 98.5 | (98.3 | - | 98.7) |  | 98.7 | (98.6 | - | 98.9) |
| Regular disinfection | 54.5 | (49.8 | - | 59.3) |  | 57.4 | (54.7 | - | 60.1) |  | 59.9 | (59.1 | - | 60.7) |  | 63.1 | (62.2 | - | 63.9) |
| Mask wearing in indoor facilities* | 99.6 | (99.4 | - | 99.8) |  | 99.8 | (99.7 | - | 99.9) |  | 99.8 | (99.7 | - | 99.9) |  | 99.7 | (99.6 | - | 99.8) |
| Mask wearing when hard keep distance* | 99.3 | (98.9 | - | 99.7) |  | 99.4 | (99.0 | - | 99.7) |  | 99.6 | (99.6 | - | 99.7) |  | 99.7 | (99.6 | - | 99.7) |
| Keeping minimal physical distance* | 94.5 | (91.8 | - | 97.2) |  | 94.9 | (93.5 | - | 96.3) |  | 96.1 | (95.8 | - | 96.5) |  | 96.4 | (96.1 | - | 96.7) |
| Refrain from visiting hospitalized patients* | 97.5 | (95.4 | - | 99.7) |  | 98.7 | (98.2 | - | 99.1) |  | 98.0 | (97.6 | - | 98.3) |  | 98.3 | (97.9 | - | 98.6) |
| Refrain from going out* | 96.7 | (94.7 | - | 98.6) |  | 98.2 | (97.5 | - | 98.9) |  | 97.8 | (97.5 | - | 98.1) |  | 97.7 | (97.4 | - | 98.1) |
| Health behavior deterioration |  |  |  |  |  |  |  |  |  |  |  |  |  |  |  |  |  |  |  |
| Decreased in physical activity† | 55.8 | (50.9 | - | 60.6) |  | 54.9 | (52.0 | - | 57.8) |  | 61.3 | (60.4 | - | 62.2) |  | 65.1 | (64.2 | - | 66.0) |
| Changes in sleep duration | 24.0 | (19.8 | - | 28.3) |  | 26.5 | (23.8 | - | 29.2) |  | 25.3 | (24.6 | - | 26.0) |  | 23.3 | (22.6 | - | 24.0) |
| Increased in consuming instant meals/soda† | 12.6 | (7.9 | - | 17.2) |  | 18.5 | (15.4 | - | 21.6) |  | 23.6 | (22.7 | - | 24.4) |  | 26.7 | (25.8 | - | 27.6) |
| Increased in consuming delivery food† | 16.4 | (11.9 | - | 20.9) |  | 28.0 | (24.6 | - | 31.3) |  | 40.5 | (39.5 | - | 41.5) |  | 47.1 | (45.9 | - | 48.4) |
| Increased in alcohol drinking† | 3.2 | (1.1 | - | 5.3) |  | 6.4 | (4.1 | - | 8.7) |  | 7.7 | (7.1 | - | 8.2) |  | 7.8 | (7.2 | - | 8.4) |
| Increased in smoking† | 21.0 | (9.4 | - | 32.7) |  | 11.4 | (7.2 | - | 15.5) |  | 9.9 | (8.2 | - | 11.5) |  | 6.5 | (4.5 | - | 8.5) |
| Abbreviations: 95% CI, 95% confidence interval *Excluded participants who responded as 'not applicable' during last 1 week †Excluded participants who responded as 'not applicable' | | | | | | | | | | | | | | | | | | | |
