# Supplementary material for: Socioeconomic inequality in compliance with precautions and health behavior changes during the COVID-19 outbreak: an analysis of the Korean Community Health Survey 2020
Source: Epidemiol Health. 2022 Jan 9;44:e2022013. doi: 10.4178/epih.e2022013 (PMC8989472; doi:10.4178/epih.e2022013)
Supplement: Supplementary Material 10. — Age-standardized rates of COVID-19 safety precautions compliance and health behavior deterioration occurrence by household income in men [file epih-44-e2022013-suppl10.docx]

| Supplementary Material 10. Age-standardized rates of COVID-19 safety precautions compliance and health behavior deterioration occurrence by household income in men | | | | | | | | | | | | | | | | | | | |
| --- | --- | --- | --- | --- | --- | --- | --- | --- | --- | --- | --- | --- | --- | --- | --- | --- | --- | --- | --- |
| COVID19-related questionnaires | **Monthly household income** | | | | | | | | | | | | | | | | | | |
|  | Q1 (lowest) | | | |  | Q2 | | | |  | Q3 | | | |  | Q4 (highest) | | | |
|  | rate, % | 95% CI | | |  | rate, % | 95% CI | | |  | rate, % | 95% CI | | |  | rate, % | 95% CI | | |
| Comply with safety precautions |  |  |  |  |  |  |  |  |  |  |  |  |  |  |  |  |  |  |  |
| Covering mouth while coughing* | 93.5 | (93.4 | - | 93.7) |  | 94.3 | (94.1 | - | 94.4) |  | 95.1 | (94.9 | - | 95.2) |  | 95.5 | (95.4 | - | 95.6) |
| Regular ventilation | 97.5 | (97.4 | - | 97.5) |  | 97.5 | (97.4 | - | 97.6) |  | 97.4 | (97.3 | - | 97.5) |  | 97.6 | (97.5 | - | 97.7) |
| Regular disinfection | 54.9 | (54.5 | - | 55.2) |  | 59.9 | (59.5 | - | 60.2) |  | 63.8 | (63.5 | - | 64.2) |  | 64.3 | (64.0 | - | 64.6) |
| Mask wearing in indoor facilities* | 99.3 | (99.2 | - | 99.4) |  | 99.4 | (99.3 | - | 99.5) |  | 99.4 | (99.4 | - | 99.4) |  | 99.6 | (99.6 | - | 99.7) |
| Mask wearing when hard keep to distance* | 98.9 | (98.8 | - | 99.0) |  | 99.1 | (99.0 | - | 99.1) |  | 99.0 | (98.9 | - | 99.0) |  | 99.1 | (99.1 | - | 99.2) |
| Keeping minimal physical distance* | 95.2 | (95.1 | - | 95.4) |  | 95.4 | (95.3 | - | 95.5) |  | 95.0 | (94.9 | - | 95.1) |  | 95.3 | (95.2 | - | 95.5) |
| Refrain from visiting hospitalized patients* | 97.6 | (97.4 | - | 97.8) |  | 98.2 | (98.0 | - | 98.3) |  | 98.2 | (98.0 | - | 98.3) |  | 98.3 | (98.2 | - | 98.4) |
| Refrain from going out* | 97.0 | (96.9 | - | 97.1) |  | 97.0 | (96.9 | - | 97.1) |  | 96.8 | (96.7 | - | 96.9) |  | 97.0 | (96.9 | - | 97.1) |
| Health behavior deterioration |  |  |  |  |  |  |  |  |  |  |  |  |  |  |  |  |  |  |  |
| Decreased in physical activity† | 51.2 | (50.8 | - | 51.6) |  | 52.6 | (52.3 | - | 53.0) |  | 53.1 | (52.7 | - | 53.5) |  | 55.0 | (54.7 | - | 55.3) |
| Changes in sleep duration | 21.4 | (21.1 | - | 21.7) |  | 18.4 | (18.1 | - | 18.7) |  | 17.3 | (17.1 | - | 17.6) |  | 17.0 | (16.7 | - | 17.2) |
| Increased in consuming instant meals/soda† | 19.8 | (19.5 | - | 20.2) |  | 21.1 | (20.8 | - | 21.4) |  | 20.0 | (19.7 | - | 20.3) |  | 21.1 | (20.8 | - | 21.4) |
| Increased in consuming delivery food† | 32.6 | (32.1 | - | 33.0) |  | 38.7 | (38.3 | - | 39.1) |  | 39.8 | (39.4 | - | 40.2) |  | 44.7 | (44.4 | - | 45.1) |
| Increased in alcohol drinking† | 8.0 | (7.7 | - | 8.2) |  | 6.9 | (6.7 | - | 7.1) |  | 6.6 | (6.4 | - | 6.8) |  | 6.3 | (6.1 | - | 6.5) |
| Increased in smoking† | 10.8 | (10.4 | - | 11.1) |  | 8.8 | (8.5 | - | 9.0) |  | 8.1 | (7.8 | - | 8.4) |  | 7.3 | (7.1 | - | 7.5) |
| Abbreviations: 95% CI, 95% confidence interval *Excluded participants who responded as 'not applicable' during last 1 week †Excluded participants who responded as 'not applicable' | | | | | | | | | | | | | | | | | | | |
