# Supplementary material for: Socioeconomic inequality in compliance with precautions and health behavior changes during the COVID-19 outbreak: an analysis of the Korean Community Health Survey 2020
Source: Epidemiol Health. 2022 Jan 9;44:e2022013. doi: 10.4178/epih.e2022013 (PMC8989472; doi:10.4178/epih.e2022013)
Supplement: Supplementary Material 11. — Age-standardized rates of COVID-19 safety precautions compliance and health behavior deterioration occurrence by household income in women [file epih-44-e2022013-suppl11.docx]

| Supplementary Material 11. Age-standardized rates of COVID-19 safety precautions compliance and health behavior deterioration occurrence by household income in women | | | | | | | | | | | | | | | | | | | |
| --- | --- | --- | --- | --- | --- | --- | --- | --- | --- | --- | --- | --- | --- | --- | --- | --- | --- | --- | --- |
| COVID19-related questionnaires | Monthly household income | | | | | | | | | | | | | | | | | | |
|  | Q1 (lowest) | | | |  | Q2 | | | |  | Q3 | | | |  | Q4 (highest) | | | |
|  | rate, % | 95% CI | | |  | rate, % | 95% CI | | |  | rate, % | 95% CI | | |  | rate, % | 95% CI | | |
| Comply with safety precautions |  |  |  |  |  |  |  |  |  |  |  |  |  |  |  |  |  |  |  |
| Covering mouth while coughing* | 95.0 | (94.9 | - | 95.1) |  | 95.2 | (95.1 | - | 95.3) |  | 96.7 | (96.6 | - | 96.8) |  | 96.9 | (96.8 | - | 97.0) |
| Regular ventilation | 98.5 | (98.3 | - | 98.6) |  | 98.4 | (98.4 | - | 98.5) |  | 98.6 | (98.5 | - | 98.7) |  | 98.5 | (98.4 | - | 98.6) |
| Regular disinfection | 56.1 | (55.7 | - | 56.5) |  | 57.9 | (57.6 | - | 58.3) |  | 61.0 | (60.6 | - | 61.4) |  | 62.3 | (61.9 | - | 62.7) |
| Mask wearing in indoor facilities* | 99.6 | (99.6 | - | 99.7) |  | 99.7 | (99.6 | - | 99.7) |  | 99.8 | (99.7 | - | 99.8) |  | 99.7 | (99.7 | - | 99.8) |
| Mask wearing when hard keep to distance* | 99.3 | (99.3 | - | 99.4) |  | 99.4 | (99.3 | - | 99.4) |  | 99.6 | (99.5 | - | 99.6) |  | 99.6 | (99.5 | - | 99.6) |
| Keeping minimal physical distance* | 96.1 | (95.9 | - | 96.3) |  | 95.9 | (95.8 | - | 96.0) |  | 96.0 | (95.8 | - | 96.1) |  | 96.2 | (96.0 | - | 96.3) |
| Refrain from visiting hospitalized patients* | 97.7 | (97.5 | - | 97.8) |  | 98.1 | (97.9 | - | 98.2) |  | 98.2 | (98.0 | - | 98.4) |  | 98.4 | (98.3 | - | 98.5) |
| Refrain from going out* | 97.9 | (97.7 | - | 98.0) |  | 97.7 | (97.5 | - | 97.8) |  | 97.9 | (97.7 | - | 98.0) |  | 97.7 | (97.6 | - | 97.9) |
| Health behavior deterioration |  |  |  |  |  |  |  |  |  |  |  |  |  |  |  |  |  |  |  |
| Decreased in physical activity† | 60.0 | (59.6 | - | 60.5) |  | 60.9 | (60.5 | - | 61.3) |  | 61.3 | (60.8 | - | 61.7) |  | 64.0 | (63.6 | - | 64.4) |
| Changes in sleep duration | 26.6 | (26.2 | - | 27.0) |  | 23.9 | (23.5 | - | 24.2) |  | 22.1 | (21.7 | - | 22.4) |  | 22.3 | (22.0 | - | 22.7) |
| Increased in consuming instant meals/soda† | 21.5 | (21.0 | - | 21.9) |  | 23.5 | (23.1 | - | 23.9) |  | 23.3 | (22.8 | - | 23.7) |  | 26.4 | (26.0 | - | 26.8) |
| Increased in consuming delivery food† | 35.6 | (35.1 | - | 36.1) |  | 39.5 | (39.1 | - | 39.9) |  | 40.6 | (40.1 | - | 41.1) |  | 46.0 | (45.5 | - | 46.5) |
| Increased in alcohol drinking† | 7.3 | (6.9 | - | 7.6) |  | 7.1 | (6.8 | - | 7.4) |  | 7.6 | (7.3 | - | 7.9) |  | 7.9 | (7.6 | - | 8.2) |
| Increased in smoking† | 12.0 | (11.4 | - | 12.6) |  | 8.5 | (8.0 | - | 9.0) |  | 7.2 | (6.6 | - | 7.8) |  | 7.3 | (6.2 | - | 8.4) |
| Abbreviations: 95% CI, 95% confidence interval *Excluded participants who responded as 'not applicable' during last 1 week †Excluded participants who responded as 'not applicable' | | | | | | | | | | | | | | | | | | | |
